# A prospective study of *XRCC1 *(X-ray cross-complementing group 1) polymorphisms and breast cancer risk

**DOI:** 10.1186/bcr1355

**Published:** 2005-11-21

**Authors:** Alpa V Patel, Eugenia E Calle, Alexandre L Pavluck, Heather Spencer Feigelson, Michael J Thun, Carmen Rodriguez

**Affiliations:** 1 Department of Epidemiology and Surveillance Research, American Cancer Society, 1599 Clifton Road NE, Atlanta, GA 30309, USA

## Abstract

**Introduction:**

The gene *XRCC1 *(X-ray repair cross-complementing group 1) encodes a protein involved in DNA base excision repair. Two non-synonymous polymorphisms in *XRCC1 *(Arg194Trp and Arg399Gln) have been shown to alter DNA repair capacity in some studies *in vitro*. However, results of previous association studies of these two *XRCC1 *variants and breast cancer have been inconsistent. We examined the association between polymorphisms in *XRCC1 *and breast cancer in the American Cancer Society Cancer Prevention Study II (CPS-II) Nutrition Cohort, a large prospective study of cancer incidence in the USA.

**Methods:**

Among the 21,965 women who were cancer-free in 1992 and gave blood between 1998 and 2001, 502 postmenopausal breast cancer cases were diagnosed between 1992 and 2001; 502 controls were matched to cases on age, race/ethnicity, and date of blood collection. Genotyping on DNA extracted from buffy coat was performed with Taqman. Conditional logistic regression was used to examine the association between each polymorphism and breast cancer risk controlling for breast cancer risk factors. We also examined whether factors associated with DNA damage, such as smoking and antioxidant intake, modified the association between *XRCC1 *polymorphisms and breast cancer.

**Results:**

We observed a significant inverse association between Trp194 carriers (Trp/Trp and Trp/Arg) compared with Trp194 non-carriers in relation to breast cancer (Arg/Arg) (odds ratio (OR) 0.62, 95% confidence interval (CI) 0.40 to 0.95). The inverse association between breast cancer and Trp194 carriers compared with non-carriers was slightly stronger among smokers (OR 0.47, 95% CI 0.24 to 0.94) than never smokers (OR 0.78, 95% CI 0.43 to 1.40). An increased risk associated with the Arg399Gln polymorphism (Gln/Gln versus Arg/Arg) was observed only among women who reported ever smoking cigarettes (OR 2.76, 95% CI 1.36 to 5.63), and not in women who were lifelong non-smokers (OR 0.64, 95% CI 0.33 to 1.26). No other factor examined modified the association between *XRCC1 *polymorphisms and breast cancer risk.

**Conclusion:**

Our results support the hypothesis that genetic variation in *XRCC1*, particularly in Arg194Trp, may influence postmenopausal breast cancer risk. In our study, genetic variation in *XRCC1 *Arg399Gln was associated with breast cancer risk only among women with a history of smoking cigarettes.

## Introduction

Base excision repair (BER) corrects localized DNA damage such as oxidized or fragmented lesions and non-bulky adducts [1]. Sources of oxidative damage include ionizing radiation and chemical carcinogens in tobacco smoke [1,2]. In the absence of this repair process, oxidized lesions and non-bulky adducts may block DNA replication or cause cytotoxic mutations and genetic instability [3]. The gene *XRCC1 *(X-ray repair cross-complementing group 1) encodes a protein involved in DNA BER that is essential in drawing different components of BER to the site of DNA damage and promoting efficiency of the BER pathway [4,5].

The *XRCC1 *gene contains 17 exons and is located on chromosome 19q13.2 [6]. Although many polymorphisms have been documented [7], two non-synonymous polymorphisms in *XRCC1 *(Arg194Trp (C→T allelic change) and Arg399Gln (G→A allelic change)) have been shown to alter DNA repair capacity in some phenotypic studies and have received considerable attention. The Trp194 variant has been associated with increased BER capacity, whereas the Gln399 variant has been associated with reduced repair capacity [8-11]. Because of its important role in the repair capacity of BER, variability in *XRCC1 *expression has been examined extensively in relation to various age-related diseases, including cancer [12]. DNA repair proficiency has also been proposed as a potential susceptibility factor for breast cancer [13].

So far, 12 observational studies have examined *XRCC1 *polymorphisms Arg194Trp and Arg399Gln in relation to breast cancer risk, with inconsistent results [7,14-24]. Some of these studies suggest that associations between *XRCC1 *polymorphisms and breast cancer risk are stronger in women who smoke or who have higher exposure to various antioxidants [7,14,24,25]. However, 8 of the 12 studies included fewer than 250 cases and had limited power to examine relationships in potentially important subgroups, such as smokers [14-17,19,21-23]. Among the remaining studies with larger sample sizes [18,20,24,25], two studies did not examine potential interactions with factors such as smoking [18,20], one reported effect modification by antioxidants, but not smoking [7,25], and the last reported a stronger association with Arg399Gln in non-smokers with detectable DNA-adduct levels [24]. Previous studies that examined the relationship between other *XRCC1 *polymorphisms and breast cancer risk generally have been null [12].

To our knowledge, only one previous study observed an association with any *XRCC1 *polymorphisms aside from Arg194Trp and Arg399Gln [19]: they observed an association with the Arg280His polymorphism in their relatively small (*n *= 250 cases) study population. Although the Arg280His polymorphism is non-synonymous, there is no functional data to support its potential role in repair capacity. A larger study (*n *= 1,000 cases) conducted by Han *et al*. [25] was unable to replicate the earlier finding, and they did not observe any association with other polymorphisms other than those at codons 194 and 399. Furthermore, they identified that most *XRCC1 *polymorphisms are all part of one haplotype block, but did not observe an association with haplotype blocks [7]. On the contrary, they observed an association with only Arg194Trp and Arg399Gln polymorphisms and breast cancer risk. Thus, in the present analysis, we examined the association between the two non-synonymous polymorphisms in *XRCC1 *that have been shown to have functional relevance in DNA repair capacity (Arg194Trp (rs1799782) and Arg399Gln (rs25487)) and postmenopausal breast cancer risk in a case-control study nested in the American Cancer Society Cancer Prevention Study II (CPS-II) Nutrition Cohort.

## Materials and methods

### Study population

Women in this analysis were drawn from the 97,786 female participants in the CPS-II Nutrition Cohort, which was established by the American Cancer Society in 1992 as a subgroup of the larger 1982 CPS-II baseline mortality cohort [26]. Most participants were aged 50 to 74 years at enrollment in 1992. At baseline they completed a 10-page self-administered questionnaire that included questions on demographic, reproductive, medical, behavioral, environmental, and dietary factors. Beginning in 1997, follow-up questionnaires were sent to cohort members every 2 years to update exposure information and to ascertain newly diagnosed cancers. Incident cancers reported on the questionnaires were verified through medical records, linkage with state cancer registries, or death certificates.

From June 1998 to June 2001, blood samples were collected from a subgroup of 39,376 cohort members (*n *= 21,965 women). After obtaining informed consent, we collected a maximum of 43 ml of non-fasting whole blood from each participant and samples were separated into aliquots of serum, plasma, red blood cells, and buffy coat. Samples then were frozen in liquid nitrogen vapor phase at about -130°C for long-term storage.

Among the 21,965 women with blood samples who were cancer-free and postmenopausal in 1992, 502 postmenopausal breast cancer cases diagnosed between 1992 and 2001 were reported through follow-up questionnaires and subsequently verified. For all cases, questionnaire information on risk factors for breast cancer was collected before the diagnosis of cancer (that is, in 1992); however, collection of DNA from buffy coat occurred in 1998 to 2001, in some cases after or only slightly before cancer diagnosis. For each case, we randomly selected one cancer-free (except non-melanoma skin cancer) female Nutrition Cohort control matched on single year of age (±6 months), race/ethnicity (white, African American, Hispanic, Asian, other/unknown), date of blood collection (within 6 months), and selected from individuals who were cancer-free (except for non-melanoma skin cancer) at the beginning of the interval preceding the diagnosis of each case using risk-set sampling [27]. The women who provided blood specimens were similar to the overall population in the distribution of most demographic and lifestyle characteristics.

### Laboratory

DNA was extracted from buffy coat and genotyping assays were performed with TaqMan (Applied Biosystems, Foster City, CA) as described previously [25]. Genotyping was performed by laboratory personnel blinded to case-control status, and 10% blind duplicates were randomly interspersed with the case-control samples to validate genotyping procedures. Concordance for the quality control samples was 100%. Overall success rate for the genotyping assays was at least 95%.

### Statistical analysis

We used a χ^2 ^test to assess whether *XRCC1 *genotype distributions were in Hardy-Weinberg equilibrium. Conditional logistic regression was used to examine the association between the *XRCC1 *polymorphisms and breast cancer in two models, one controlling for matching factors only and the other controlling for matching factors and other possible confounders.

Conditional logistic regression modeling was performed because controls were individually matched at the time of selection to each case patient. Potential confounders included in the multivariate models included education (up to and including high school graduate, some college, college graduate or higher), age at menopause (less than 45, 45 to 54, 55 or more years, unknown) and menarche (less than 12/missing, 12 or more years), parity (nulliparous, 1 to 2, 3 or more, unknown), age at first live birth (less than 25, 25 or more, unknown), personal history of benign breast disease (yes, no), family history of breast cancer in mother or sisters (yes, no), alcohol intake (none, not more than one drink per day, more than one drink per day, unknown), postmenopausal hormone replacement use (never, current estrogen replacement therapy, current combination estrogen–progestin replacement therapy, former estrogen replacement therapy, former estrogen–progestin replacement therapy, unknown), smoking status (never, ever, unknown), body mass index (less than 25, 25 to less than 30, 30 or more, unknown), and physical activity (metabolic equivalents (MET-hours) per week) (less than 7, 7 to less than 17.5, 17.5 to less than 31.5, 31.5 or more, unknown). With the exception of age at menopause (8.5% missing), all covariates had less than 3.5% values unknown.

To test whether factors proposed to be associated with oxidative stress modified the association between *XRCC1 *polymorphisms and breast cancer risk, we constructed multiplicative interaction terms with cigarette smoking, fruit and vegetable intake, alcohol intake, physical activity, body mass index, folate intake, and multivitamin use. We also tested for potential interaction between the two polymorphisms of interest. Statistical interaction was assessed in multivariate models using the likelihood ratio test, and *p *< 0.05 was considered statistically significant [28].

## Results

Genotyping data were missing on 6 cases and 9 controls for Arg194Trp and on 27 cases and 23 controls for Arg399Gln. All genotypes were in Hardy–Weinberg equilibrium. Cases and controls were largely white (99%), with a median age of 62 years (range 43 to 75) at enrollment in 1992. Table 1 shows the genotype frequencies, odds ratios (ORs) and 95% confidence intervals (CIs) for the association between *XRCC1 *polymorphisms and breast cancer. Because the Trp194 allele is uncommon (the allele frequency among controls was 7.4%), logistic regression models combined the Trp194 carriers (Trp/Trp and Arg/Trp genotypes combined) and compared them with non-carriers (Arg/Arg). After adjustment for various factors, breast cancer risk was 38% lower among Trp194 carriers than among non-carriers (OR 0.62, 95% CI 0.40 to 0.95). Risk was 27% higher among women who were homozygous for the Gln399 variant than among women homozygous for the wild-type (Arg) allele, although this was not statistically significant (OR 1.27, 95% CI 0.79 to 2.02; Table 1).

We hypothesized *a priori *that the association between *XRCC1 *polymorphisms and breast cancer risk might be stronger in women who had experienced greater exposure to DNA-damaging agents, such as tobacco smoke. Carriers of Trp194 were at lower risk of breast cancer than Trp194 non-carriers regardless of smoking status, although the inverse association was slightly attenuated among lifelong non-smokers (ever smokers OR 0.47, 95% CI 0.24 to 0.94; never smokers OR 0.78, 95% CI 0.43 to 1.40; interaction *p *= 0.28; Table 2). For the Arg399Gln polymorphism, results differed by smoking status (interaction *p *= 0.01). Among women who had ever smoked cigarettes, the OR was 2.76 when comparing the Gln/Gln399 genotype with the Arg/Arg399 genotype (95% CI 1.36 to 5.63), whereas no increased risk was associated with this polymorphism among lifelong non-smokers (OR 0.64, 95% CI 0.33 to 1.26; Table 2).

The association between *XRCC1 *polymorphisms and breast cancer risk did not differ by other environmental factors examined (data not shown). The association between *XRCC1 *polymorphisms and breast cancer risk also did not differ by age at diagnosis or stage of disease (data not shown). Finally, when assessing the combined effects of both *XRCC1 *polymorphisms on breast cancer risk, women with 'low-risk' genotypes for both polymorphisms (Trp194 carriers and Gln399 non-carriers) had a 49% lower incidence of breast cancer than women with 'high-risk' genotypes for both polymorphisms (Trp194 non-carriers and Gln399 carriers; 95% CI 0.28 to 0.90).

## Discussion

Results from this study support the hypothesis that genetic variation in *XRCC1 *may affect a woman's breast cancer risk, especially in women who have ever smoked cigarettes. Overall, women carrying the Trp194 allele had a 38% lower incidence of breast cancer than women who were Trp194 non-carriers. This association persisted regardless of smoking history. In contrast, the relationship between the Arg399Gln polymorphism and breast cancer risk seemed to be limited to women who had ever smoked. Among ever smokers, women with the Gln/Gln399 genotype had a 2.76-fold higher risk of breast cancer than women with the Arg/Arg399 genotype; however, we did not observe an increased risk among women who had never smoked.

Our findings are consistent with those from four previous studies of *XRCC1 *polymorphisms and breast cancer risk that reported an inverse association between the Trp194 carriers and breast cancer risk in mostly Caucasian [7,14] and other [15,22] ethnic populations. Other previous studies observed a borderline positive [16,17,23] or no [19,24] association between the Trp194 carriers and breast cancer risk. The Arg399Gln polymorphism has been associated with breast cancer risk in only two studies [15,23], whereas most studies observed no overall association [7,14,16-22,24].

*XRCC1 *Arg194Trp and Arg399Gln polymorphisms have been shown to affect *XRCC1 *protein-product expression and to alter BER capacity [8-11]. Oxidative stress caused by cigarette smoking can induce oxidative DNA damage; minor variations in DNA repair capacity may therefore more significantly influence risk in subpopulations that experience greater DNA damage, such as smokers. Polymorphisms in *XRCC1 *have been associated with risk of many smoking-related cancers such as lung, bladder, and esophageal cancer [12]. However, few studies have examined the relationship between *XRCC1 *polymorphisms and breast cancer risk in subgroups of the population that may experience higher levels of DNA damage, such as smokers. Only two [14,24] of four [7,14,20,24] previous studies that examined *XRCC1 *variation by smoking reported evidence of effect modification by smoking status. Duell *et al*. [14] reported a stronger association for Gln/Gln399 genotype with increasing duration of smoking, whereas Shen *et al*. [24] observed a stronger association among women who had never smoked but had detectable levels of DNA adducts. They also reported stronger associations for Gln/Gln399 than for Arg/Arg399 in subjects with a high intake of antioxidants or fruit/vegetables [24]. Other studies reported weaker inverse associations with the Trp194 allele or stronger positive associations with Gln/Gln399 genotype and breast cancer risk among women who drank alcohol [15], had a positive family history of breast cancer [17], or had high plasma folate levels [7]. However, studies that examined the relationship between *XRCC1 *polymorphisms and breast cancer did not observe any meaningful differences by factors such as family history [24], alcohol intake [20,24], reproductive factors [18,23], ionizing radiation [14,20], and body mass index [18,20,24].

The primary limitation of our study is the limited statistical power to examine whether factors such as the duration of smoking modify the association between breast cancer and the polymorphisms of interest. The statistical power is limited by both small sample size and low frequency of smokers in our cohort. Although the association observed between *XRCC1 *polymorphisms and breast cancer risk among smokers in this study is interesting and adds support to the hypothesis, we cannot exclude chance as a possible explanation for the findings. For this reason, further replication of the findings is needed in larger study populations with higher smoking prevalence. Strengths of our study include the prospective collection of questionnaire data, which eliminates the potential for selection bias, and the extensive information collected from questionnaires on various factors that may be associated with oxidative DNA damage.

## Conclusion

Our results suggest that non-synonymous coding polymorphisms in *XRCC1 *may be associated with postmenopausal breast cancer risk, especially among women who have ever smoked. Additional studies are needed to examine the association of these polymorphisms with breast cancer risk, focusing on subpopulations that may experience greater exposure to DNA-damaging agents.

## Abbreviations

BER = base excision repair; CI = confidence interval; CPS-II = Cancer Prevention Study II; OR = odds ratio; *XRCC1 *= X-ray cross-complementing group 1.

## Competing interests

The authors declare that they have no competing interests.

## Authors' contributions

AVP was the principal investigator, developed the analytic plan, and drafted the manuscript. EEC participated in study design, conception of the study hypotheses, and critical review of the manuscript. ALP performed statistical analyses. HSF, MJT, and CR contributed to the analytic plan and were involved in critical review of the manuscript. All authors read and approved the final manuscript.

## Figures and Tables

**Table 1:** Conditional logistic regression models examining the association between *XRCC1 *polymorphisms and postmenopausal breast cancer risk

Polymorphism	No. of cases	No. of controls	OR (95% CI)^a^	OR (95% CI)^b^
Arg194Trp (485 pairs)				
Arg/Arg	437	415	1.00 (reference)	1.00 (reference)
Arg/Trp or Trp/Trp^c^	48	70	0.66 (0.45–0.97)	0.62 (0.40–0.95)
Arg399Gln (452 pairs)				
Arg/Arg	196	194	1.00 (reference)	1.00 (reference)
Arg/Gln	195	202	0.95 (0.71–1.27)	1.01 (0.73–1.39)
Gln/Gln	61	56	1.08 (0.71–1.63)	1.27 (0.79–2.02)
Arg/Gln or Gln/Gln	256	258	0.98 (0.75–1.28)	1.06 (0.78–1.44)

^a^Odds ratio and corresponding 95% confidence interval adjusted for matched pairs; ^b^multivariate odds ratio and corresponding 95% confidence interval adjusted for: matched pairs, body mass index, age at menopause, age at menarche, alcohol intake, education, parity, age at first live birth, physical activity, history of breast cysts, family history of breast cancer in mother or sister, use of hormone replacement therapy, and smoking status; ^c^homozygote TT group = one case and two controls. *XRCC1*, X-ray cross-complementing group 1.

**Table 2:** Conditional logistic regression models examining *XRCC1 *polymorphisms and postmenopausal breast cancer stratified by smoking status.

Polymorphism	Never smokers	Ever smokers
Arg194Trp		
Arg/Arg		
No. of cases/no. of controls	217/237	216/175
OR^a ^(95% CI)	1.00 (ref.)	1.00 (ref.)
Arg/Trp or Trp/Trp		
No. of cases/no. of controls	26/36	21/32
OR^a ^(95% CI)	0.78 (0.43–1.40)	0.47 (0.24–0.94)
	Interaction *p *= 0.28	
Arg399Gln		
Arg/Arg		
No. of cases/no. of controls	107/104	88/88
OR^a ^(95% CI)	1.00 (ref.)	1.00 (ref.)
Arg/Gln		
No. of cases/no. of controls	100/112	92/89
OR^a ^(95% CI)	0.91 (0.57–1.43)	1.06 (0.66–1.72)
Gln/Gln		
No. of cases/no. of controls	23/35	38/20
OR^a ^(95% CI)	0.64 (0.33–1.26)	2.76 (1.36–5.63)
	Interaction *p *= 0.01	

^a^Multivariate odds ratio and corresponding 95% confidence interval adjusted for: matched pairs, body mass index, age at menopause, age at menarche, alcohol intake, education, parity, age at first live birth, physical activity, history of breast cysts, family history of breast cancer in mother or sister, use of hormone replacement therapy, and smoking status. *XRCC1*, X-ray cross-complementing group 1.
